# Supplementary material for: Downregulating carnitine palmitoyl transferase 1 affects disease progression in the SOD1 G93A mouse model of ALS
Source: Commun Biol. 2021 Apr 30;4:509. doi: 10.1038/s42003-021-02034-z (PMC8087699; doi:10.1038/s42003-021-02034-z)
Supplement: Supplementary file 1 — Description of Additional Supplementary Files [file 42003_2021_2034_MOESM1_ESM.docx]

Description of Additional Supplementary Files

Title: Supplementary Data 1a-f

Description: Significant changes in abundance of microbiome communities at phyla, family, and genus level. Differential abundance testing using DESeq2 with the Benjamin-Hochberg adjustment. p-adj = adjusted p-value. Log2FC =Log2 fold change. SOD1=SOD1 G93A mice. WT=wildtype mice, SOD1Cpt1a/Cpt1a =SOD1 G93A mice with a Cpt1a P479L homozygote mutation, E=Etomoxir, P=Placebo, HFD=High fat diet, CORT=Corticosterone.

Title: Supplementary Data 2

Description: Source data for figure 1, 2, 5-9.
