# Supplementary material for: Downregulating carnitine palmitoyl transferase 1 affects disease progression in the SOD1 G93A mouse model of ALS
Source: Commun Biol. 2021 Apr 30;4:509. doi: 10.1038/s42003-021-02034-z (PMC8087699; doi:10.1038/s42003-021-02034-z)
Supplement: Supplementary file 2 — Supplementary Information [file 42003_2021_2034_MOESM2_ESM.pdf]

Supplementary information

Downregulating carnitine palmitoyl transferase 1 affects disease progression in the SOD1 G93A mouse model of ALS

Michael Sloth Trabjerg[1], Dennis Christian Andersen[1], Pam Huntjens[1], Kirsten Egelund Oklinski[1], Luise Bolther[1], Jonas Laugård Hald[1], Amalie Elton Baisgaard[1], Kasper Mørk[1], Nikolaj Warming[1], Ulla Bismark Kullab[1], Lona John Kroese[2], Colin Eliot Jason Pritchard[2], Ivo Johan Huijbers[2], John Dirk Vestergaard Nieland[1]*

[1] Department of Health Science and Technology, Aalborg University, 9220 Aalborg, Denmark

[2] Mouse Clinic for Cancer and Aging Research, Transgenic Facility, The Netherlands Cancer Institute, Amsterdam, 1066, Netherlands

* Corresponding author

Supplementary Figure 1: Behavioral effects of downregulating CPT1 activity by etomoxir from day 100

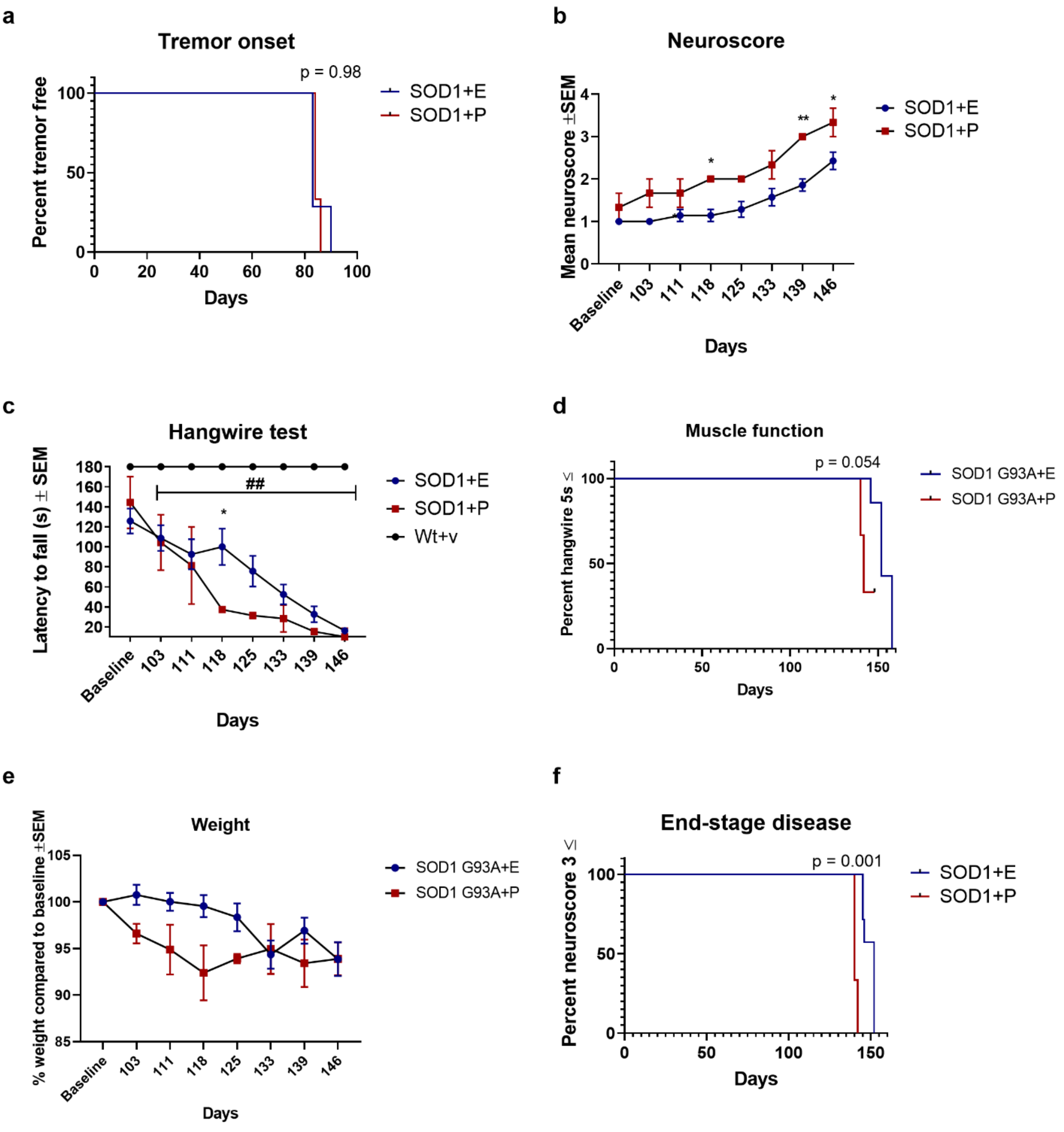

a) Tremor onset was defined as first sign of onset of disease and was evaluated by visual inspection. **b)** Neuroscore was evaluated once a week from day 90 (baseline). Mean Neuroscore ± SEM. **c)** Latency to fall of the hangwire grid test was evaluated once a week. Mean latency to fall of ± SEM. **d)** Muscle function. Defined as a latency to fall from the hangwire test 5s ≥. **e)** Mean weight at indicated timepoints ± SEM. **f)** End stage disease was defined as neuroscore 3 ≤. Treatment was initiated at day 100 and administered daily by oral gavage (5mg/kg). Experiment was performed once. Data analyses was performed by using log-rank tests (a, d, f) or repeated-measure two-way ANOVA

followed by Tukey post hoc test. N = 3 – 7. * $p \leq 0.05$, ** $p \leq 0.01$ between SOD1+P and SOD1+E. ## $p \leq 0.01$ between SOD1 and Wt+v. SOD1; SOD1 G93A genotype. Wt; Wild-type. E; etomoxir. P; Placebo. V; Vehicle.

Supplementary Figure 2: Sex does not affect disease severity at day 140 or survival in SOD1 G93A mice

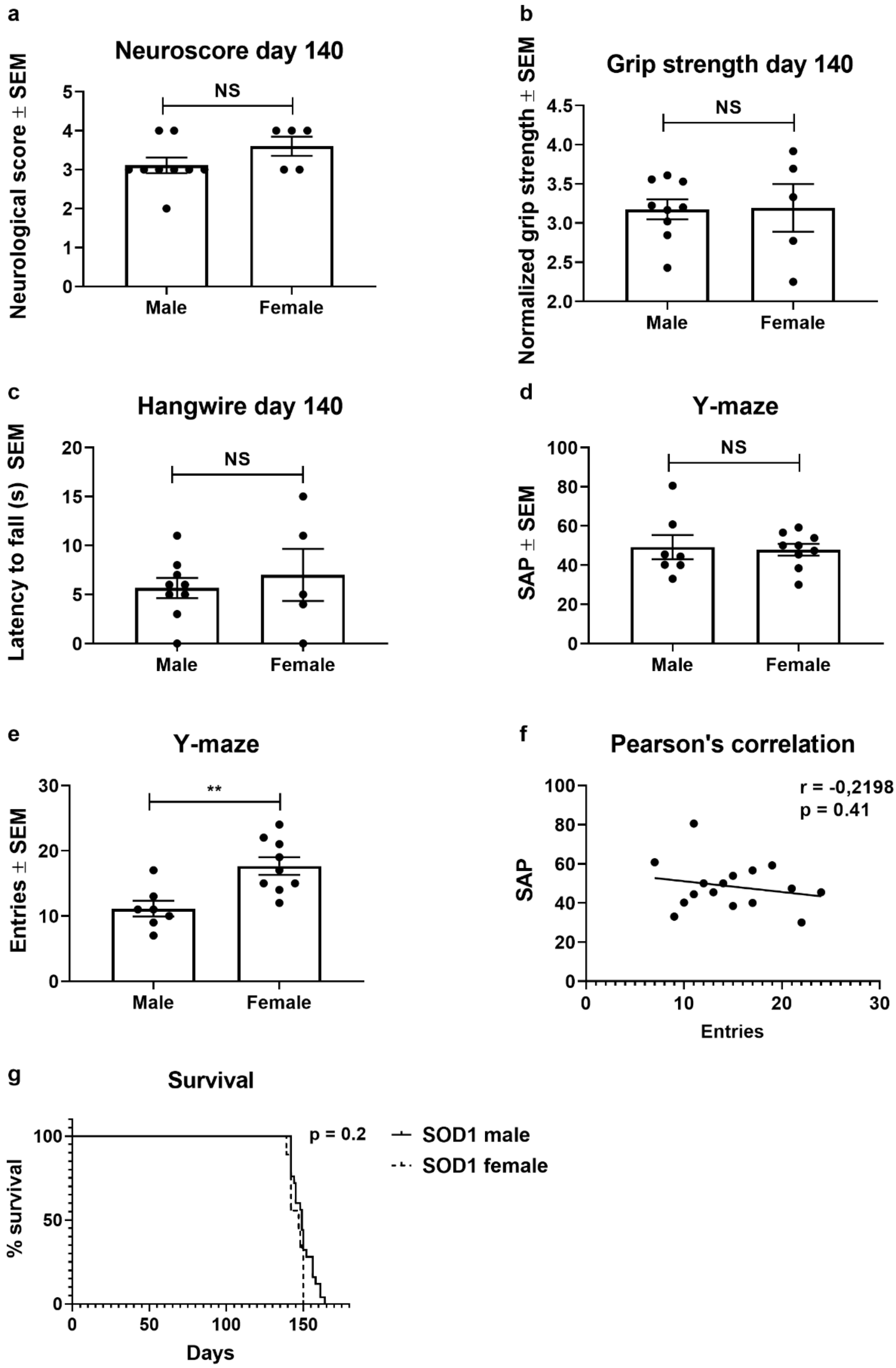

a) Neuroscore was evaluated at day 140. Mean neurological score ± SEM. **b)** Grip strength was evaluated and normalized to weight. Mean normalized strength ± SEM. **c)** Latency to fall of the

hangwire grid test was evaluated at day 140. Mean latency to fall of ± SEM. **d)** Visiospatial memory was evaluated by y-maze test ay day 140. Mean spontaneous alternation percentage ± SEM. **e)** Mean number of entries ± SEM in the y-maze test at day 140. **f)** Pearson correlation test between number of entries and spontaneous alternation percentage. **g)** Survival was defined as a neuroscore below four. All statistical differences was evaluated by unpaired t-test or log-rank test (g). N = 5 – 9. * $p \leq 0.05$, ** $p \leq 0.01$ between SOD1 male and SOD1+ female. NS=Non-significant, SEM=Standard error of the mean, SOD1=SOD1 G93A genotype.

Supplementary Figure 3: Mean fold change gene expression in brain tissue from SOD1 G93A mice

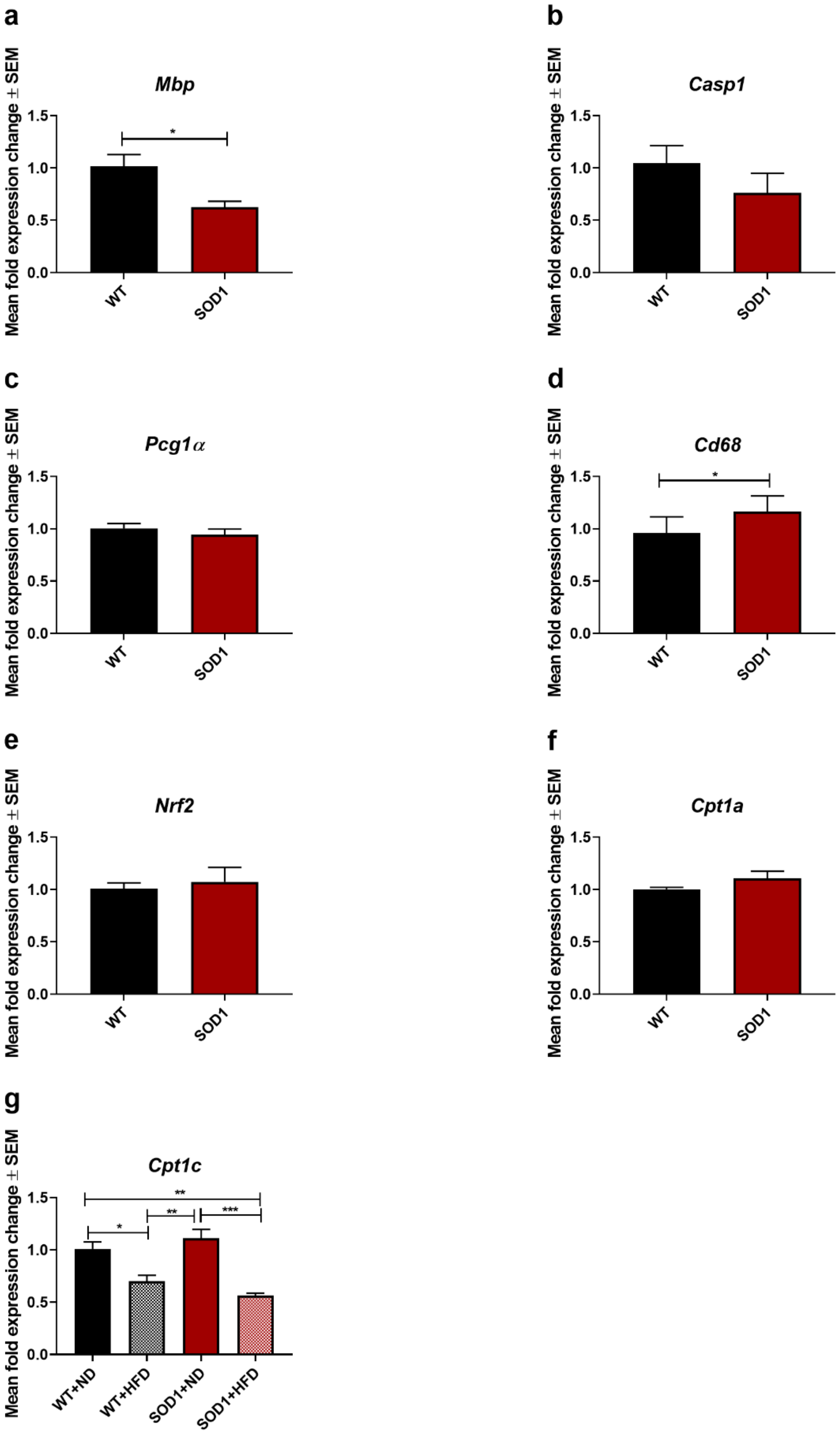

a) *Mbp* gene expression in brain tissue from SOD1 and Wt mice. **b)** *Casp1* gene expression in brain tissue from SOD1 and Wt mice. **c)** *Pcg1α* gene expression in brain tissue from SOD1 and Wt mice . **d)** *Cd68* gene expression in brain tissue from SOD1 and Wt mice. **e)** *Nrf2* gene expression in brain tissue from SOD1 and Wt mice. **f)** *Cpt1a* gene expression in brain tissue from SOD1 and Wt mice. **g)** *Cpt1c* gene expression in brain tissue from HFD experiment. Tissues were harvested at day 145 – 150. n=3-4, error bars represent the standard error of the mean (SEM) fold change. Data are representative of one RT-qPCR experiment based on one animal experiment. Data was analyzed by unpaired t-test or one-way ANOVA followed by Tukey post hoc test. Gene expression was normalized to *β-actin* and *Hprt*. $*p \leq 0.05$; $**p \leq 0.01$; $***p \leq 0.001$; $****p \leq 0.0001$. WT= wildtype, SOD1= SOD1 G93A genotype. ND=normal diet, HFD= High fat diet.

Supplementary Figure 4: Immunohistochemistry staining for MBP, GFAP and CPT1A in the lumbar spinal cord

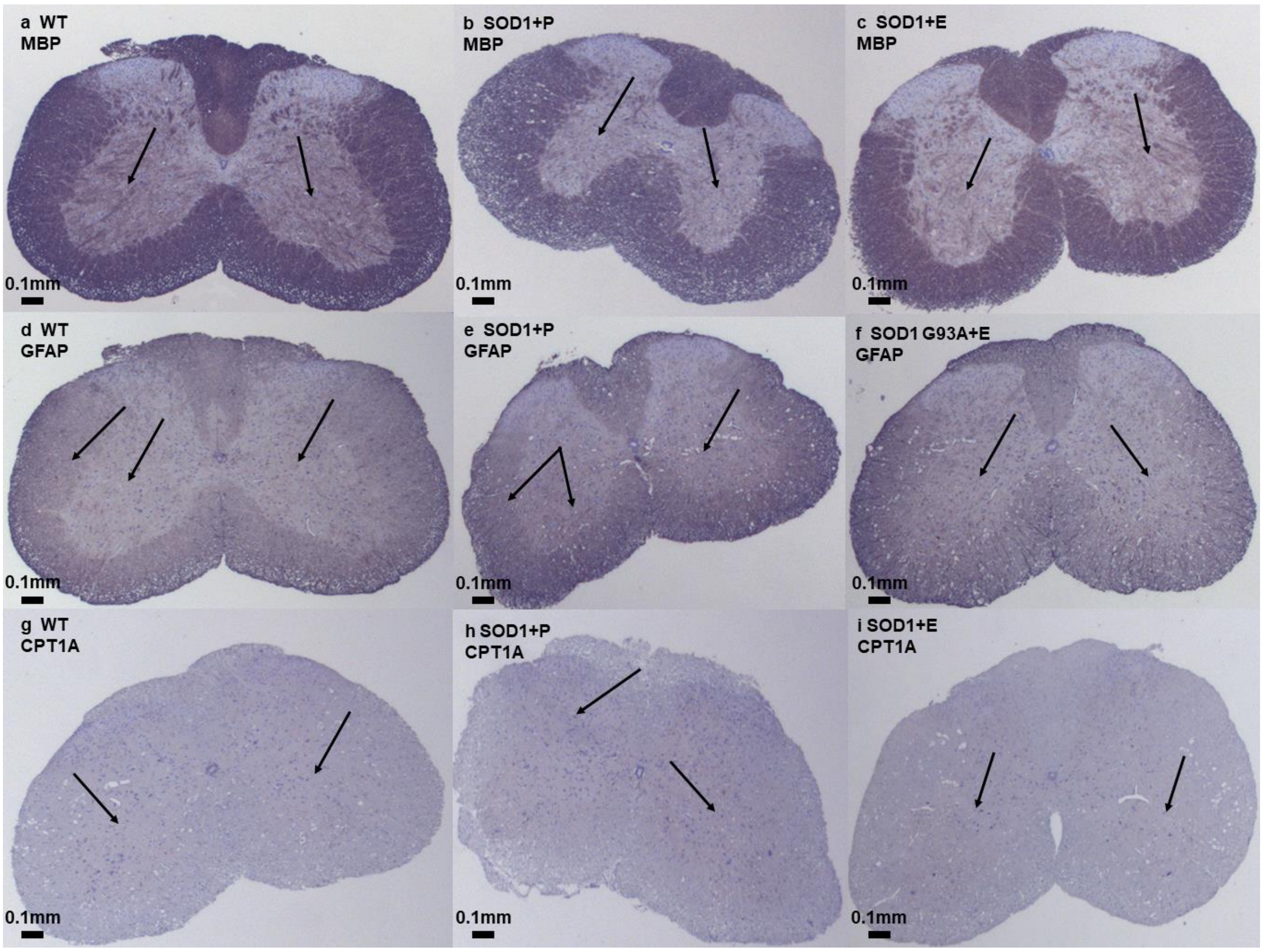

a-c) MBP staining in lumbar spinal cord from Wt, SOD1+P and SOD1+E mice at day 130 indicating decreased labeling in SOD1+P mice (arrows). **d-f)** GFAP staining in lumbar spinal cord from Wt, SOD1+P and SOD1+E mice at day 130 indicating increased labeling in SOD1+P mice (arrows). **g-i)** CPT1A staining in lumbar spinal cord from Wt, SOD1+P and SOD1+E mice at day 130 indicating increased labeling in SOD1+P mice (arrows). All images are presented with 2.5x magnification. N = 2 – 4 animals per group. WT=wild-type, SOD1 = SOD1 G93A genotype, E=etomoxir, P=placebo.

Supplementary Figure 5: α-diversity measures in fecal samples analyzed by 16s rRNA sequencing

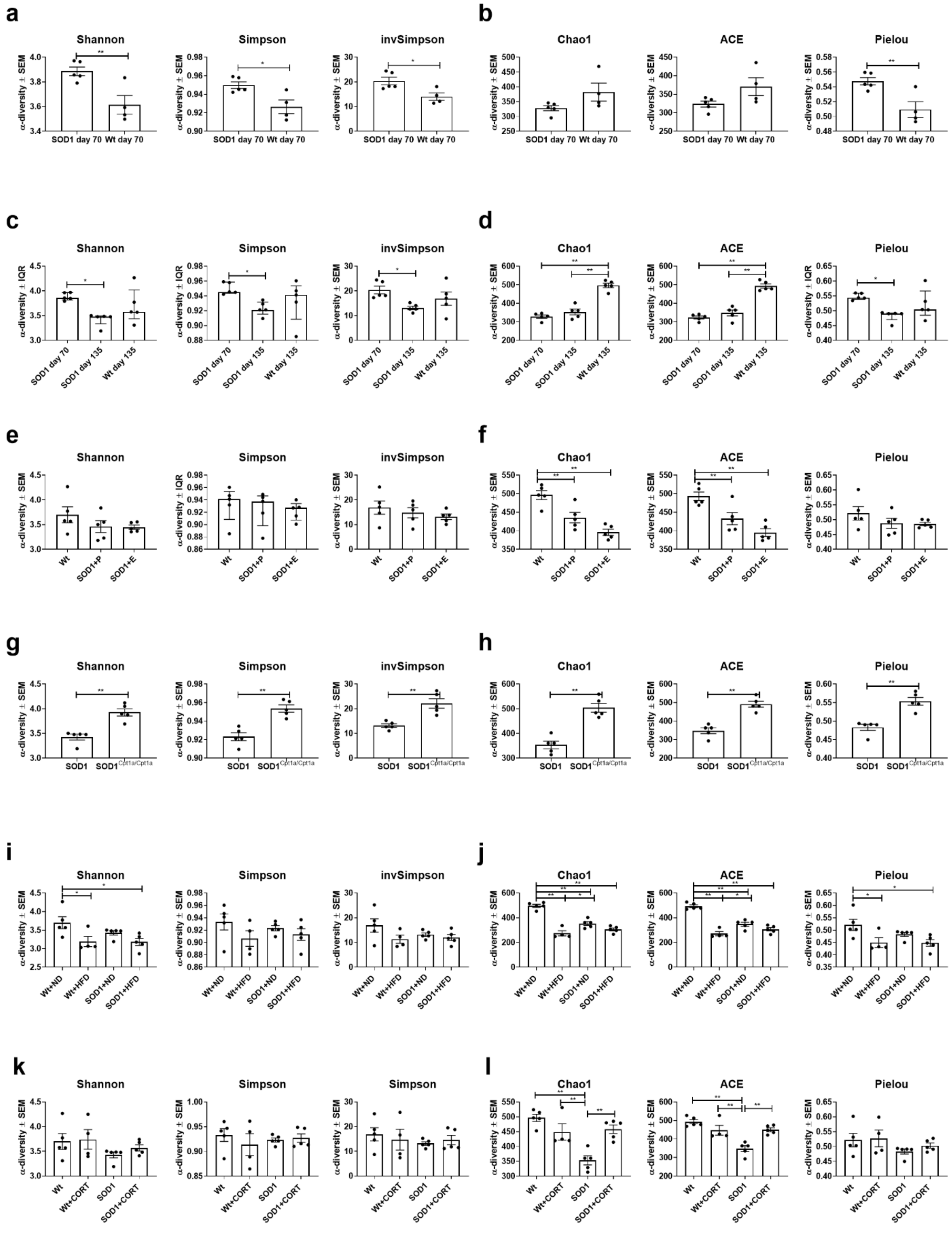

a) α-diversity measures (Shannon, Simpson and invSimpson) in SOD1 day 70 and Wt mice. **b)** α-diversity measures (Chao1, ACE and Pielou) in SOD1day 70 and Wt mice. **c)** α-diversity measures (Shannon, Simpson and invSimpson) in SOD1 day 70, SOD1 day 130 and Wt mice day 130, **d)** α-diversity measures (Chao1, ACE and Pielou) in SOD1 day 70, SOD1 day 130 and Wt mice day 130. **e)** α-diversity measures (Shannon, Simpson and invSimpson) in SOD1+P, SOD1+E and Wt mice day 130. **f)** α-diversity measures (Chao1, ACE and Pielou) in SOD1+P, SOD1+E and Wt mice day 130, **g)** α-diversity measures (Shannon, Simpson and invSimpson) in SOD1 and SOD1$^{Cpt1a/Cpt1a}$ mice day 130. **h)** α-diversity measures (Chao1, ACE and Pielou) in SOD1 and SOD1$^{Cpt1a/Cpt1a}$ mice day 130. **i)** α-diversity measures (Shannon, Simpson and invSimpson) in SOD1+HFD, SOD1, Wt+HFD and Wt mice day 130 day. **j)** α-diversity measures (Chao1, ACE and Pielou) in SOD1+HFD, SOD1, Wt+HFD and Wt mice day 130 day. **k)** α-diversity measures (Shannon, Simpson and invSimpson) in SOD1+CORT, SOD1, Wt+CORT and Wt mice day 130. **l)** α-diversity measures (Chao1, ACE and Pielou) in SOD1+CORT, SOD1, Wt+CORT and Wt mice day 130. Fecal pellet samples were harvested at day 70 or day 130, n = 4-5. Data are representative of one 16S rRNA sequencing experiment. Wt=wildtype, SOD1=SOD1 G93A genotype, SOD1$^{Cpt1a/Cpt1a}$=SOD1 G93A mice with homozygote *Cpt1a p479l* mutation. E=etomoxir, P=placebo, HFD=high fat diet, CORT=corticosterone. Shannon=Shannon index, invSimpson=Inverse Simpson index, Chao1=Chao1 index, ACE=ACE measure of species richness, Pielou= Pielou's measure of species evenness.

Supplementary Figure 6: Principal component analysis indicates inter-group differences between genotypes and interventions (β-diversity)

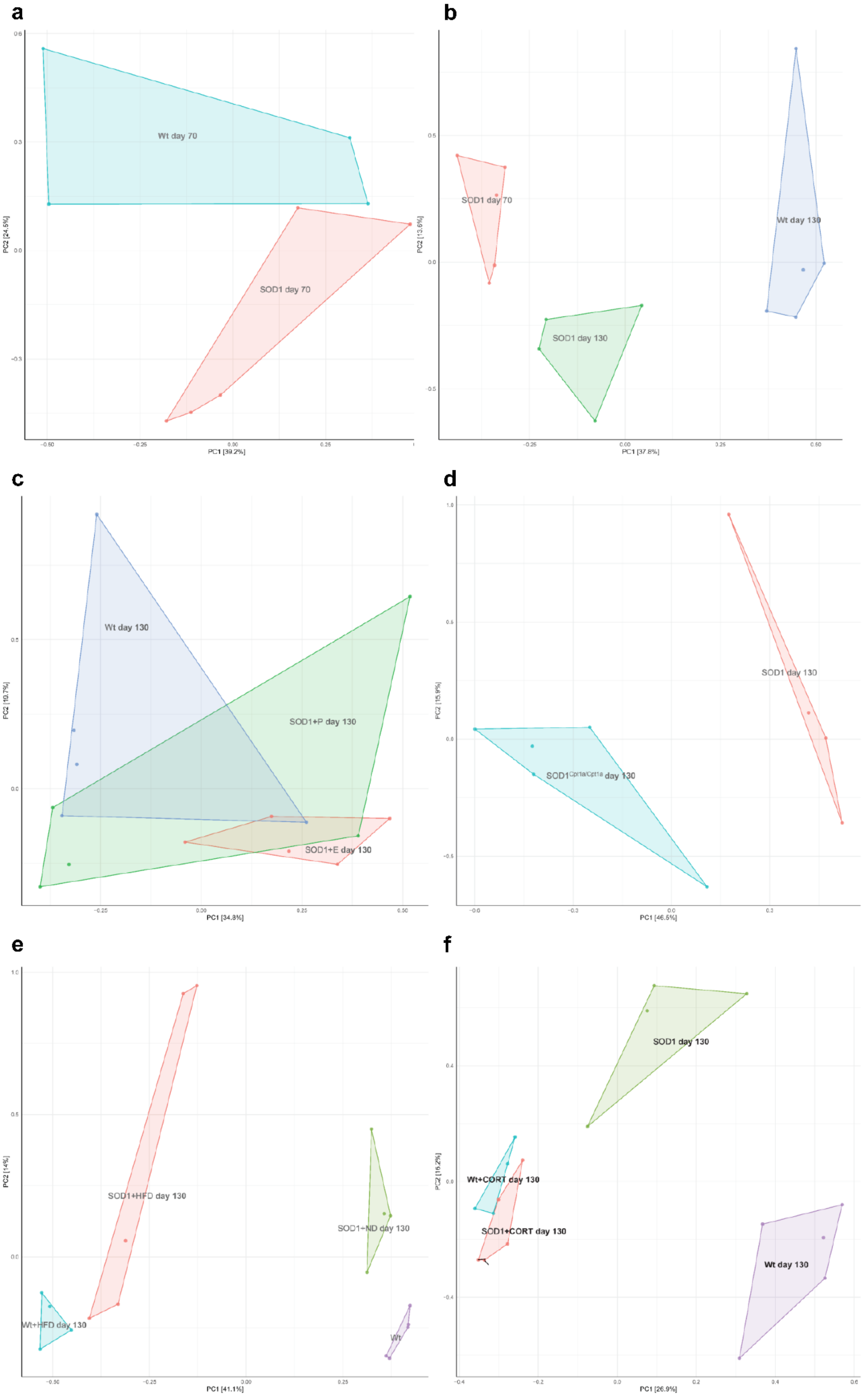

a) PCA with Hellinger transformation on fecal microbiome samples from Wt and SOD1 G93A day 70 samples. **b)** PCA with Hellinger transformation on fecal microbiome samples from SOD1 G93A day 70, SOD1 G93A day 130 and Wt day 130 samples. **c)** PCA with Hellinger transformation on fecal microbiome samples from SOD1 G93A+E, SOD1 G93A+P and Wt mice day 130 samples. **d)** PCA with Hellinger transformation on fecal microbiome samples from SOD1 G93A and SOD1 G93A$^{Cpt1a/Cpt1a}$ mice day 130 samples. **e)** PCA with Hellinger transformation on fecal microbiome samples from SOD1 G93A, SOD1+HFD, Wt+HFD and Wt mice day 130 samples. **f)** PCA with Hellinger transformation on fecal microbiome samples from SOD1 G93A, SOD1+CORT, Wt+CORT and Wt mice day 130 samples. Prior to the multivariate statistics analyses (PCA), OTU's that are not present in more than 0.1% relative abundance in any sample have been removed. The data has been transformed initially by applying the hellinger transformation (Legendre & Gallagher, 2001). The relative contribution (eigenvalue) of each axis to the total inertia in the data is indicated in percent at the axis titles. WT=wildtype, SOD1 G93A = SOD1 G93A genotype, SOD1 G93A$^{Cpt1a/Cpt1a}$ = SOD1 G93A mice with homozygote *Cpt1a P479L* mutation. E=etomoxir, P=placebo, HFD=High fat diet, CORT=Corticosterone. PCA=Principal Component Analysis.

Supplementary Figure 7: Most abundant microbial communities at the phyla level

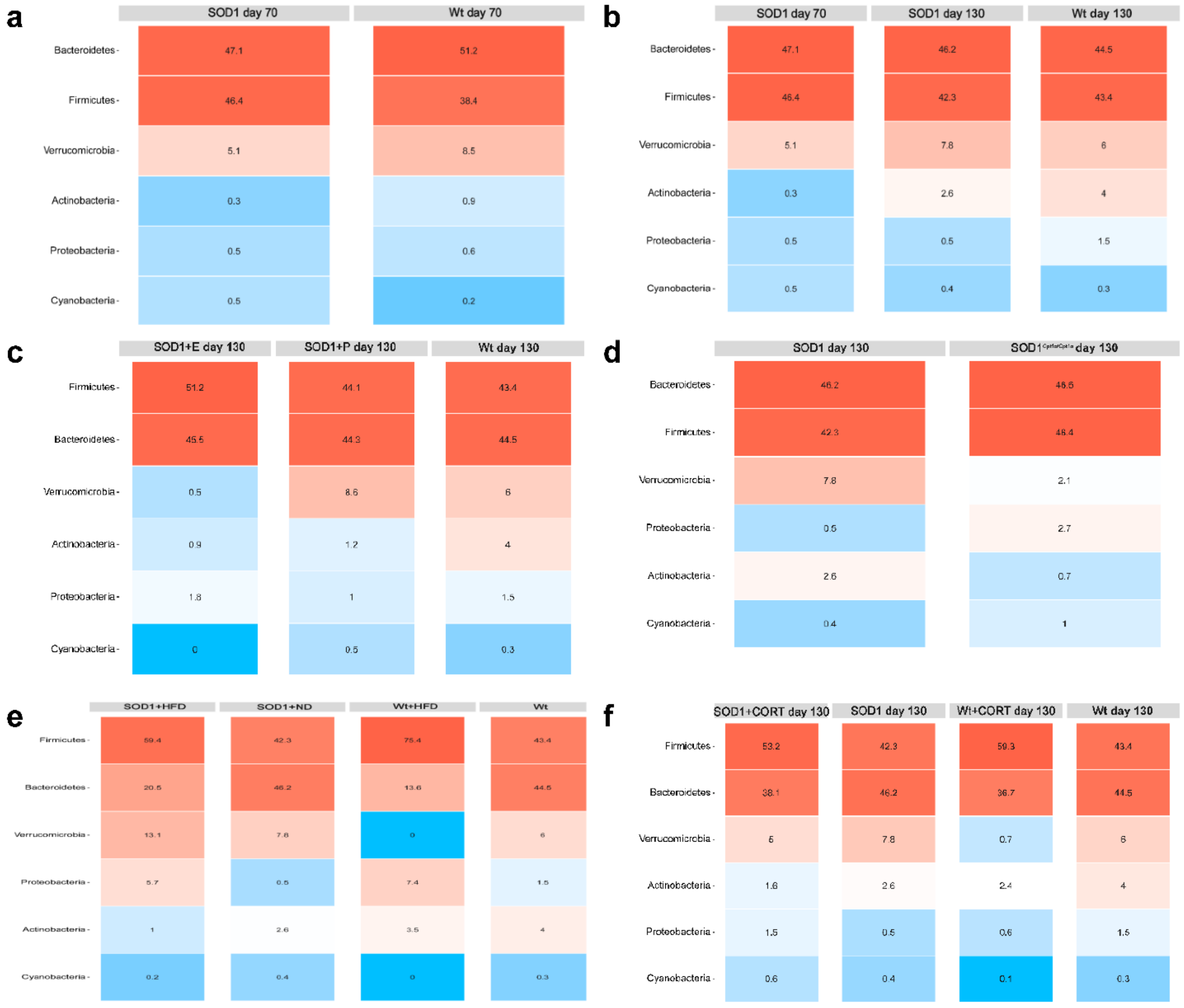

a) Heatmap illustrating the 6 most abundant communities at the phyla level in fecal samples from SOD1 mice and Wt mice at day 70. Values represents mean relative abundance. **b)** Heatmap illustrating the 6 most abundant communities at the phyla level in fecal samples from SOD1 mice at day 70, day 130 and Wt mice at day 130. Values represents mean relative abundance. **c)** Heatmap illustrating the 6 most abundant communities at the phyla level in fecal samples from SOD1+P, SOD1+E and Wt mice at day 130. Values represents mean relative abundance. **d)** Heatmap illustrating the 6 most abundant communities at the phyla level in fecal samples from SOD1 and SOD1$^{Cpt1a/Cpt1a}$ mice at day 130. Values represents mean relative abundance. **e)** Heatmap illustrating the 6 most abundant communities at the phyla level in fecal samples from SOD1+ND, SOD1+HFD, Wt+ND and Wt+HFD mice at day 130. Values represents mean relative abundance. **f)** Heatmap illustrating the 6 most abundant communities at the phyla level in fecal samples from SOD1, SOD1+CORT, Wt and Wt+CORT mice at day 130. Values represents mean relative abundance. Fecal pellet samples were harvested at day 70 or day 130, n = 4-5. Data are representative of one 16S rRNA sequencing experiment. Wt=wildtype, SOD1=SOD1 G93A genotype, SOD1$^{Cpt1a/Cpt1a}$=SOD1 G93A mice with homozygote *Cpt1a p479l* mutation. E=etomoxir, P=placebo, HFD=high fat diet, CORT=corticosterone.

Supplementary Figure 8: Most abundant microbial communities at the family level

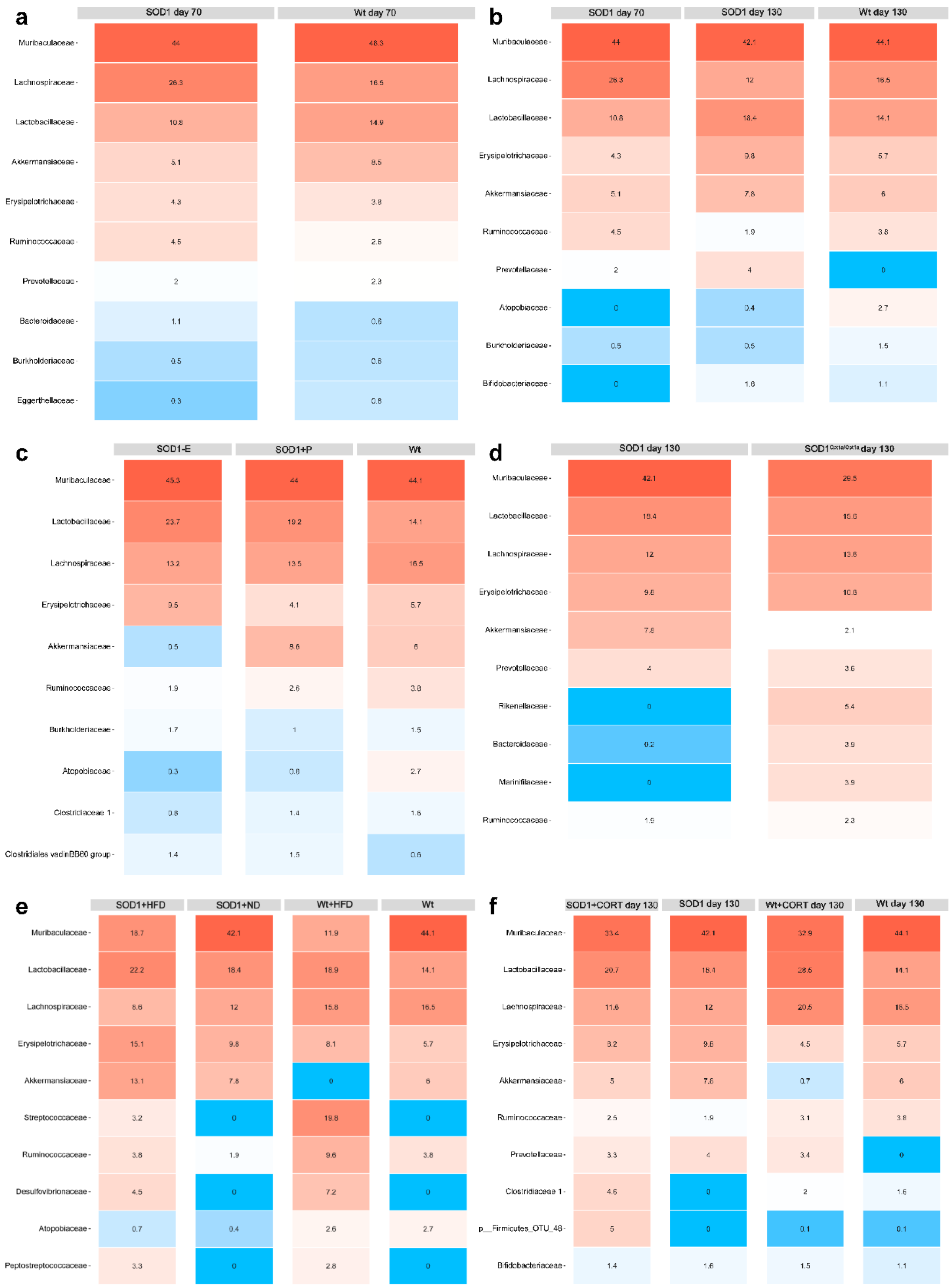

a) Heatmap illustrating the 10 most abundant communities at the family level in fecal samples from SOD1 mice and Wt mice at day 70. Values represents mean relative abundancy. **b)** Heatmap illustrating the 10 most abundant communities at the family level in fecal samples from SOD1 mice day 70, day 130 and Wt mice day 130. Values represents mean relative abundancy. **c)** Heatmap illustrating the 10 most abundant communities at the family level in fecal samples from SOD1+P, SOD1+E and Wt mice at day 130. Values represents mean relative abundancy. **d)** Heatmap illustrating the 10 most abundant communities at the family level in fecal samples from SOD1 and SOD1$^{Cpt1a/Cpt1a}$ mice at day 130. Values represents mean relative abundancy. **e)** Heatmap illustrating the 10 most abundant communities at the phyla level in fecal samples from SOD1+ND, SOD1+HFD, Wt+ND and Wt+HFD mice at day 130. Values represents mean relative abundancy. **f)** Heatmap illustrating the 10 most abundant communities at the family level in fecal samples from SOD1, SOD1+CORT, Wt and Wt+CORT mice at day 130. Values represents mean relative abundancy. Fecal pellet samples were harvested at day 70 or day 130, n = 4-5. Data are representative of one 16S rRNA sequencing experiment. Wt=wildtype, SOD1=SOD1 G93A genotype, SOD1$^{Cpt1a/Cpt1a}$=SOD1 G93A mice with homozygote *Cpt1a p479l* mutation. E=etomoxir, P=placebo, HFD=high fat diet, CORT=corticosterone.
