# Supplementary material for: Downregulating carnitine palmitoyl transferase 1 affects disease progression in the SOD1 G93A mouse model of ALS
Source: Commun Biol. 2021 Apr 30;4:509. doi: 10.1038/s42003-021-02034-z (PMC8087699; doi:10.1038/s42003-021-02034-z)
Supplement: Supplementary file 5 — Reporting Summary [file 42003_2021_2034_MOESM5_ESM.pdf]

nature research

Corresponding author(s): John Dirk Vestergaard Nieland

Last updated by author(s): Mar 9, 2021

Reporting Summary

Nature Research wishes to improve the reproducibility of the work that we publish. This form provides structure for consistency and transparency in reporting. For further information on Nature Research policies, see our Editorial Policies and the Editorial Policy Checklist.

Statistics

For all statistical analyses, confirm that the following items are present in the figure legend, table legend, main text, or Methods section.

n/a	Confirmed	
☐	☒	The exact sample size (n) for each experimental group/condition, given as a discrete number and unit of measurement
☐	☒	A statement on whether measurements were taken from distinct samples or whether the same sample was measured repeatedly
☐	☒	The statistical test(s) used AND whether they are one- or two-sided<br>*Only common tests should be described solely by name; describe more complex techniques in the Methods section.*
☒	☐	A description of all covariates tested
☐	☒	A description of any assumptions or corrections, such as tests of normality and adjustment for multiple comparisons
☐	☒	A full description of the statistical parameters including central tendency (e.g. means) or other basic estimates (e.g. regression coefficient) AND variation (e.g. standard deviation) or associated estimates of uncertainty (e.g. confidence intervals)
☐	☒	For null hypothesis testing, the test statistic (e.g. F, t, r) with confidence intervals, effect sizes, degrees of freedom and P value noted<br>*Give P values as exact values whenever suitable.*
☒	☐	For Bayesian analysis, information on the choice of priors and Markov chain Monte Carlo settings
☒	☐	For hierarchical and complex designs, identification of the appropriate level for tests and full reporting of outcomes
☒	☐	Estimates of effect sizes (e.g. Cohen's d, Pearson's r), indicating how they were calculated

Our web collection on statistics for biologists contains articles on many of the points above.

Software and code

Policy information about availability of computer code

Data collection	Not relevant
Data analysis	Not relevant

For manuscripts utilizing custom algorithms or software that are central to the research but not yet described in published literature, software must be made available to editors and reviewers. We strongly encourage code deposition in a community repository (e.g. GitHub). See the Nature Research guidelines for submitting code & software for further information.

Data

Policy information about availability of data

All manuscripts must include a data availability statement. This statement should provide the following information, where applicable:

- Accession codes, unique identifiers, or web links for publicly available datasets
- A list of figures that have associated raw data
- A description of any restrictions on data availability

Source data for the microbiome studies are freely available from Mendeley Data using http://dx.doi.org/10.17632/6zt29bjbzd.1. Source data for figures are available in Supplementary Data 2. In addition, data that support the findings of this study are available from the corresponding author (J.D.V.N) upon reasonable request.

Field-specific reporting

Please select the one below that is the best fit for your research. If you are not sure, read the appropriate sections before making your selection.

- [x] Life sciences
- [ ] Behavioural & social sciences
- [ ] Ecological, evolutionary & environmental sciences

For a reference copy of the document with all sections, see nature.com/documents/nr-reporting-summary-flat.pdf

Life sciences study design

All studies must disclose on these points even when the disclosure is negative.

Sample size	Sample size was estimated based on previous studies and a pilot study described in the manuscript.
Data exclusions	We performed Grubbs test to evaluate potential outliers. If outliers were present, they were removed.
Replication	Animal experiments were succesful replicated minimum twice. Repeated experiments produced comparable results and findings were considered reproducible.
Randomization	Mice were randomized into cages before baseline assesments.
Blinding	Investigators were blinded during animal experiments, data collection and video analyses as described in the method section.

Reporting for specific materials, systems and methods

We require information from authors about some types of materials, experimental systems and methods used in many studies. Here, indicate whether each material, system or method listed is relevant to your study. If you are not sure if a list item applies to your research, read the appropriate section before selecting a response.

Materials & experimental systems

n/a	Involved in the study	
[ ]	[x]	Antibodies
[x]	[ ]	Eukaryotic cell lines
[x]	[ ]	Palaeontology and archaeology
[ ]	[x]	Animals and other organisms
[x]	[ ]	Human research participants
[x]	[ ]	Clinical data
[x]	[ ]	Dual use research of concern

Methods

n/a	Involved in the study	
[x]	[ ]	ChIP-seq
[x]	[ ]	Flow cytometry
[x]	[ ]	MRI-based neuroimaging

Antibodies

Antibodies used	The following primary antibodies were used: MBP 1:800 (Abcam, CAT# ab7349), GFAP 1:1000 (Agilent, CAT# Z033429-02), CPT1A 1:400 (ThermoFisher Scientific, CAT# PA5-69347), IBA-1 1:500 (Wako, CAT#013-27691), CHAT 1:400 (Merck Millipore, CAT#ab144p). The following secondary HRP-conjugated antibodies were used: Goat Anti-rat 1:500 (ThermoFisher Scientific, CAT# PA1-84708), and Goat Anti-rabbit 1:500 (Dako, CAT# P0448).
Validation	All antibodies were validated by the manufacture of in previously published papers.

Animals and other organisms

Policy information about studies involving animals; ARRIVE guidelines recommended for reporting animal research

Laboratory animals	The study involved:<br>Species; mus musculus<br>Strains;<br>10 week old C57bl/6J female mice<br>10 week old B6.Cg-Tg(SOD1*G93A)1Gur/J female mice<br>10 week old B6.Cg-Tg(SOD1*G93A)1gur/J x B6J-Cpt1a<em1Nki> female mice
Wild animals	The study did not involve wild animals
Field-collected samples	The study did not involve field-collected samples.
Ethics oversight	All experiments were approved by the Danish Animal Experiment Inspectorate and followed the National and European guidelines for

conducting animal experiments. Animal experiments was conducted according to the ARRIVE guidelines.

Note that full information on the approval of the study protocol must also be provided in the manuscript.
